# Synthesis and *In Vitro* Antimicrobial Evaluation of New 1,3,4-Oxadiazoles Bearing 5-Chloro-2-methoxyphenyl Moiety

**DOI:** 10.1155/2013/725673

**Published:** 2013-03-31

**Authors:** Basavapatna N. Prasanna Kumar, Kikkeri N. Mohana, Lingappa Mallesha, Kikkeri P. Harish

**Affiliations:** ^1^Department of Studies in Chemistry, University of Mysore, Manasagangotri, Mysore 570 006, India; ^2^The Postgraduate Department of Chemistry, JSS College of Arts, Commerce and Science, Ooty Road, Mysore 25, India

## Abstract

A series of new 1,3,4-oxadiazole derivatives, **4(a–h)**, containing 5-chloro-2-methoxy benzohydrazide moiety were synthesized by the reaction of 5-chloro-2-methoxybenzoate with different aromatic carboxylic acids. These newly synthesized compounds were characterized by FT-IR, ^1^H NMR, mass spectra, and also by elemental analysis. All the newly synthesized compounds were screened for their antibacterial and antifungal activities. Antimicrobial studies revealed that compounds **4c**, **4f**, and **4g** showed significant activity against tested strains.

## 1. Introduction

Resistance to number of antimicrobial agents among a variety of clinically significant bacteria is becoming increasingly important. There are various problems arising with the use of antimicrobials such as local tissue irritation, interference with wound healing process, hypersensitivity reactions, system toxicity, narrow antimicrobial spectrum, and emergency of resistance [1]. So, the increasing clinical importance of drug resistant microbial pathogens has additional urgency in microbiological and antifungal research. A wide variety of heterocyclic systems have been explored for developing pharmaceutically important molecules. Among them the derivatives of oxadiazoles have been playing an important role in the medicinal chemistry [2]. The 1,3,4-oxadiazole derivatives have been found to exhibit diverse biological activities such as antimicrobial [3, 4], anti HIV [5], antitubercular [6], antimalarial [7], anti-inflammatory [8, 9], anticonvulsant [10], and antitumor [11]. The 2,5-disubstituted-1,3,4-oxadiazole derivatives are known for various pharmacological activities such as antibacterial [12], antihypertensive [13], anticonvulsant [14], and antiproliferative [15]. The choice of 1,3,4-oxadiazole is due to its multiapplicability in the field of medicine. In the present study, some new 1,3,4-oxadiazoles **4(a–h)** have been synthesized and characterized by different spectral studies. All the new compounds were screened for their antibacterial and antifungal studies.

## 2. Results and Discussion

### 2.1. Chemistry

**Scheme 1:**
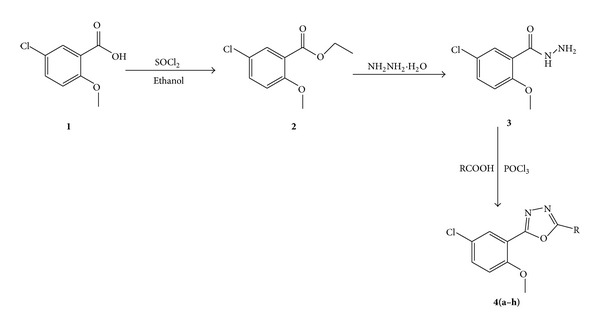
Scheme for synthesis of the new oxadiazoles **4(a–h)**.

The novel 1,3,4-oxadiazoles **4(a–h)** were synthesized according to Scheme 1. Formation of 1,3,4-oxadiazole derivatives, **4(a–h),** was confirmed by recording their elemental analyses, FT-IR, ^1^H NMR, and mass spectra. The absorptions around 3050 cm^−1^ in synthesized compounds confirm the aromatic C–H stretching vibrations and the appearance of a medium to strong absorption bands above 1600 cm^−1^ due to a stretching vibration of the azomethine (C=N) bond formation in synthesized compound. The ^1^H NMR spectra of **4c** showed that singlet in the region of *δ*, 3.99–3.90, is due to the three protons of the methoxy groups. The mass spectra of **4c** showed molecular ion peak at *m*/*z* 317.0 which is in agreement with the molecular formula, C_16_H_13_ClN_2_O_3_. The elemental analyses data showed good agreement between the experimentally determined values and the theoretically calculated values within ±0.4%. The chemical structures and physical data of all the synthesized compounds are tabulated in Table 1.

### 2.2. *In Vitro* Antimicrobial Activity

The antibacterial activity of compounds **4(a–h)** was evaluated and compared with bacteriomycin and gentamycin as standard drug (Table 2). All the tested compounds showed antibacterial activity against four pathogenic bacterial strains. Among the series, **4(a–h)**, compound **4c** exhibited an elevated antibacterial activity against tested bacterial strains. Compounds **4f** and **4g** showed good antibacterial activity against all the tested organisms. Compounds **4h**,** 4b**,** 4a**,** 4d,** and **4e** showed moderate inhibitory activity. 

The *in vitro* antifungal activity of the synthesized compounds **4(a–h)** was studied against *Fusarium oxysporum*. The results were compared with the standard drug nystatin as in Table 2. Compounds **4c**, **4f,** and **4g** showed good antifungal activity, when compared with other compounds in the series against *F. oxysporum*. Compounds **4h**,** 4b**,** 4a**,** 4d,** and **4e** were found to be moderately active against tested fungal strain. 

In the present study, different electron withdrawing and electron donating groups attached to oxadaizole ring as substituents were linke to benzene ring. The close survey of antimicrobial efficacy indicated that the inhibition values of all the compounds exhibited a varied range of antibacterial and antifungal activities against all the tested microbial strains. The electron donating methoxy group in **4c** showed good antimicrobial activity against tested microbial strains. The methoxy group and electron withdrawing fluorine atom in **4f** and **4g** produce enhanced antimicrobial activity. Compounds **4a**, **4b**,** 4d**, **4e** and **4h** exhibited moderate activity when compared **4c**, **4f** and **4g**. The above studies reveal that the nature of the linkage (substituent on aromatic ring) influences the antimicrobial activity. Among the compounds, **4(a–h)** showed antimicrobial activity in the order of **4c** > **4f** > **4g** > **4h** > **4b > 4a > 4d** > **4e** against tested bacterial and fungal strains. 

## 3. Conclusion

In conclusion, series of new 1,3,4-oxadiazoles, **4(a–h)**, were synthesized in good yield and were characterized by different spectral studies and their antimicrobial activity has been evaluated. Compounds **4c**, **4f,** and **4g** produced significant changes in activity against tested microbial strain. Therefore, this work presents a potent, wide-spectrum antimicrobial activity of the compounds. The nature of functional linkage and substituents (electron withdrawing and electron donating groups) on benzene ring is crucial for antimicrobial activities.

## 4. Experimental

### 4.1. Chemistry

Melting range was determined by Veego Melting Point VMP-III apparatus. Elemental analyses were recorded on VarioMICRO superuser V1.3.2 Elementar. The FT-IR spectra were recorded using KBr discs on FT-IR Jasco 4100 infrared spectrophotometer. ^1^H NMR spectra were recorded on Bruker DRX-500 spectrometer at 400 MHz using CDCl_3_ as solvent and TMS as an internal standard. Mass spectral data were obtained by LC/MSD Trap XCT. All solvents and reagents were purchased from Sigma-Aldrich Chemicals Pvt. Ltd.

#### 4.1.1. Synthesis of Ethyl 5-Chloro-2-methoxybenzoate (**2**)

5-Chloro-2-methoxybenzoic acid (**1**) was converted into ethyl 5-chloro-2-methoxybenzoate (**2**) by the esterification reaction as per the reported procedure[16, 17]. The mixture of 5-chloro-2-methoxybenzoic acid (**1**, 0.01 mmol) was taken in ethanol (10 mL), and thionyl chloride (0.015 mmol) was added slowly and cooled to 5–10°C. Then the reaction mass was heated to reflux for 2 hr. The reaction mass was concentrated through rotavapor under reduced pressure. The residue was dissolved in dichloromethane and washed with water. The organic layer was concentrated under reduced pressure to get the product. Yield: 76%; mp 97–99°C.

#### 4.1.2. Synthesis of 5-Chloro-2-methoxybenzohydrazide (**3**)

Compound **2** was converted into 5-chloro-2-methoxybenzohydrazide (**3**), by reacting with hydrazine hydrate in ethanol medium as per the reported procedure [18, 19]. To a mixture of ethyl 5-chloro-2-methoxybenzoate (**2**, 0.01 mmol) and ethanol (10 mL) at 0–5°C, hydrazine hydrate (0.02 mmol) was added. The reaction mass was heated to reflux for 6 hr. The reaction completion was monitored by TLC. The reaction mixture was concentrated to half volume. The solid obtained was filtered and washed with ethanol. The obtained solid was dried to get the pure product. Yield: 74%; mp 144–146°C.

#### 4.1.3. General Procedure for the Synthesis of 1,3,4-Oxadiazole Derivatives **4**(**a–h**)

An equimolar mixture of acid hydrazide (**3**, 0.2 mmol) with different aromatic carboxylic acids (0.0011 mmol) was refluxed with phosphorous oxychloride (5 volume with respect to the weight of compound **3**). The mixture was refluxed at 100°C for 4 hr. The completion of the reaction was confirmed by the thin layer chromatography (TLC). After completion, the reaction mass was cooled to room temperature and quenched with ice cold water and stirred for 1 hr. The solid obtained was filtered and washed with water. Then recrystallised with ethanol and dried to get the pure product. 

*Synthesis of 2-(5-Chloro-2-methoxyphenyl)-5-(4-fluorophenyl)-1,3,4-oxadiazole* (***4a***). White solid. FT-IR (KBr, cm^−1^): 3070 (Ar C–H), 1615 (C=N), 1589 (C=C), 1076 (C–O stretch of oxadiazole ring). ^1^H-NMR (400 MHz, CDCl_3_): *δ* 8.09 (d, 2H), 7.98 (s, 1H), 7.47 (d, 2H), 7.01 (d, 2H), 4.01 (s, 3H). MS (ESI) *m*/*z*: 304 (M^+^). Elemental analysis found (calculated) for C_15_H_10_ClFN_2_O_2_ (%): C, 59.22 (59.13); H, 3.40 (3.31); N, 9.02 (9.19).

*Synthesis of 2-(5-Chloro-2-methoxyphenyl)-5-(4-nitrophenyl)-1,3,4-oxadiazole* (***4b***). Yellow solid. FT-IR (KBr, cm^−1^): 3060 (Ar C–H), 1655 (C=N), 1576 (C=C), 1054 (C–O stretch of oxadiazole ring). ^1^H-NMR (400 MHz, CDCl_3_): *δ* 8.43 (d, 2H), 8.36 (d, 2H), 8.04 (s, 1H), 7.54–7.05 (d, 2H), 4.02 (s, 3H). MS (ESI) *m*/*z*: 331 (M^+^). Elemental analysis found (calculated) for C_15_H_10_ClN_3_O_4_ (%): C, 54.22 (54.31); H, 3.02 (3.04); N, 12.70 (12.67).

*Synthesis of 2-(5-Chloro-2-methoxyphenyl)-5-(4-methoxyphenyl)-1,3,4-oxadiazole* (***4c***). White solid. FT-IR (KBr, cm^−1^): 3070 (Ar C–H), 1675 (C=N), 1576 (C=C), 1054 (C–O stretch of oxadiazole ring). ^1^H-NMR (400 MHz, CDCl_3_): *δ* 8.09 (d, 2H), 7.99 (s, 1H), 7.47 (d, 2H), 7.01–7.04 (d, 2H), 3.99 (s, 3H), 3.9 (s, 3H). MS (ESI) *m*/*z*: 316 (M^+^). Elemental analysis found (calculated) for C_16_H_13_ClN_2_O_3_ (%): C, 60.50 (60.67); H, 4.20 (4.14); N, 8.66 (8.84). 

*Synthesis of 2-(5-Chloro-2-methoxyphenyl)-5-(4-chlorophenyl)-1,3,4-oxadiazole* (***4d***). White solid. FT-IR (KBr, cm^−1^): 3070 (Ar C–H), 1655 (C=N), 1580 (C=C), 1070 (C–O stretch of oxadiazole ring). ^1^H-NMR (400 MHz, CDCl_3_): *δ* 8.19 (d, 2H), 7.85 (s, 1H), 7.58 (d, 2H), 7.25–7.22 (d, 2H), 4.0 (s, 3H). MS (ESI) *m*/*z*: 321 (M^+^). Elemental analysis found (calculated) for C_15_H_10_Cl_2_N_2_O_2_ (%): C, 56.15 (56.10); H, 3.05 (3.14); N, 8.80 (8.72).

*Synthesis of 2-(5-Chloro-2-methoxyphenyl)-5-(4-bromophenyl)-1,3,4-oxadiazole* (***4e***). White solid. FT-IR (KBr, cm^−1^): 3050 (Ar C–H), 1650 (C=N), 1570 (C=C), 1040 (C–O stretch of oxadiazole ring). ^1^H-NMR (400 MHz, CDCl_3_): *δ* 8.01 (d, 2H), 7.88 (s, 1H), 7.56 (d, 2H), 7.29–7.27 (d, 2H), 3.95 (s, 3H). MS (ESI) *m*/*z*: 365 (M^+^). Elemental analysis found (calculated) for C_15_H_10_BrClN_2_O_2_ (%): C, 48.20 (49.28); H, 2.66 (2.76); N, 7.60 (7.66). 

*Synthesis of 2-(5-Chloro-2-methoxyphenyl)-5-(2-fluoro-3-methoxyphenyl)-1,3,4-oxadiazole* (***4f***). Off-white solid. FT-IR (KBr, cm^−1^): 3070 (Ar C–H), 1670 (C=N), 1570 (C=C), 1060 (C–O stretch of oxadiazole ring). ^1^H-NMR (400 MHz, CDCl_3_): *δ* 7.89 (s, 1H), 7.80 (s, 1H), 7.43 (d, 2H), 7.29 (d, 1H), 7.17 (m, 1H), 3.90 (s, 3H), 3.88 (s, 3H). MS (ESI) *m*/*z*: 334 (M^+^). Elemental analysis found (calculated) for C_16_H_12_ClFN_2_O_3_ (%): C, 57.20 (57.41); H, 3.40 (3.61); N, 8.40 (8.37).

*Synthesis of 2-(5-Chloro-2-methoxyphenyl)-5-(2-fluoro-5-methoxyphenyl)-1,3,4-oxadiazole* (***4g***). Off white solid. FT-IR (KBr, cm^−1^): 3060 (Ar C–H), 1665 (C=N), 1550 (C=C), 1050 (C–O stretch of oxadiazole ring). ^1^H-NMR (400 MHz, CDCl_3_): *δ* 7.94 (s, 1H), 7.80 (d, 2H), 7.43 (d, 2H), 7.07 (s, 1H), 4.0 (s, 3H), 3.95 (s, 3H). MS (ESI) *m*/*z*: 334 (M^+^). Elemental analysis found (calculated) for C_16_H_12_ClFN_2_O_3_ (%): C, 57.30 (57.41); H, 3.52 (3.61); N, 8.40 (8.37).

*Synthesis of 2-(5-Chloro-2-methoxyphenyl)-5-(2,6-difluorophenyl)-1,3,4-oxadiazole* (***4h***). White solid. FT-IR (KBr, cm^−1^): 3050 (Ar C–H), 1675 (C=N), 1585 (C=C), 1070 (C–O stretch of oxadiazole ring). ^1^H-NMR (400 MHz, CDCl_3_): *δ* 8.05 (s, 1H), 7.80 (d, 2H), 7.43 (d, 2H), 7.20 (m, 1H), 3.9 (s, 3H). MS (ESI) *m*/*z*: 322 (M^+^). Elemental analysis found (calculated) for C_15_H_9_ClF_2_N_2_O_2_ (%): C, 55.70 (55.83); H, 2.72 (2.81); N, 8.50 (8.68).

### 4.2. Antibacterial Activity

Antibacterial activity of the synthesized compounds was determined against Gram-positive bacteria (*Bacillus subtilis* MTCC 121 and *Staphylococcus aureus* MTCC 7443) and Gram-negative bacteria (*Xanthomonas campestris* MTCC 7908 and *Escherichia coli* MTCC 7410) in DMF by disc diffusion method on nutrient agar medium [20]. The sterile medium (nutrient agar medium, 15 mL) in each Petri plate was uniformly smeared with cultures of Gram-positive and Gram-negative bacteria. Sterile discs of 10 mm diameter (HiMedia) were placed in the Petri plates, to which 50 *µ*L (1 mg/mL: i.e., 50 *µ*g/disc) of the different synthesized compounds was added. The treatments also included 50 *µ*L of DMF as negative, bacteriomycin and gentamycin as positive control for comparison. For each treatment, three replicates were maintained. The plates were incubated at 37 ± 2°C for 24 h and the zone of inhibition was determined. 

### 4.3. Antifungal Activity

The synthesized compounds were screened for their antifungal activity against *Fusarium oxysporum* MTCC 2480 in DMF by poisoned food technique [21]. Potato dextrose agar (PDA) medium was prepared and about 15 mL of PDA was poured into each Petri plate and allowed to solidify. Five mm disc of seven-day old culture of the test fungi was placed at the center of the Petri plates and incubated at 26°C for 7 days. After incubation, the percentage inhibition was measured and three replicates were maintained for each treatment. Nystatin was used as standard. All the synthesized compounds were tested (at the dosage of 500 *µ*L of the novel compounds/Petri plate, where concentration was 0.1 mg/mL) by poisoned food technique. 

## Figures and Tables

**Table 1:** Chemical structure and physical data of 1,3,4-oxadiazoles **4(a**–**h)**.

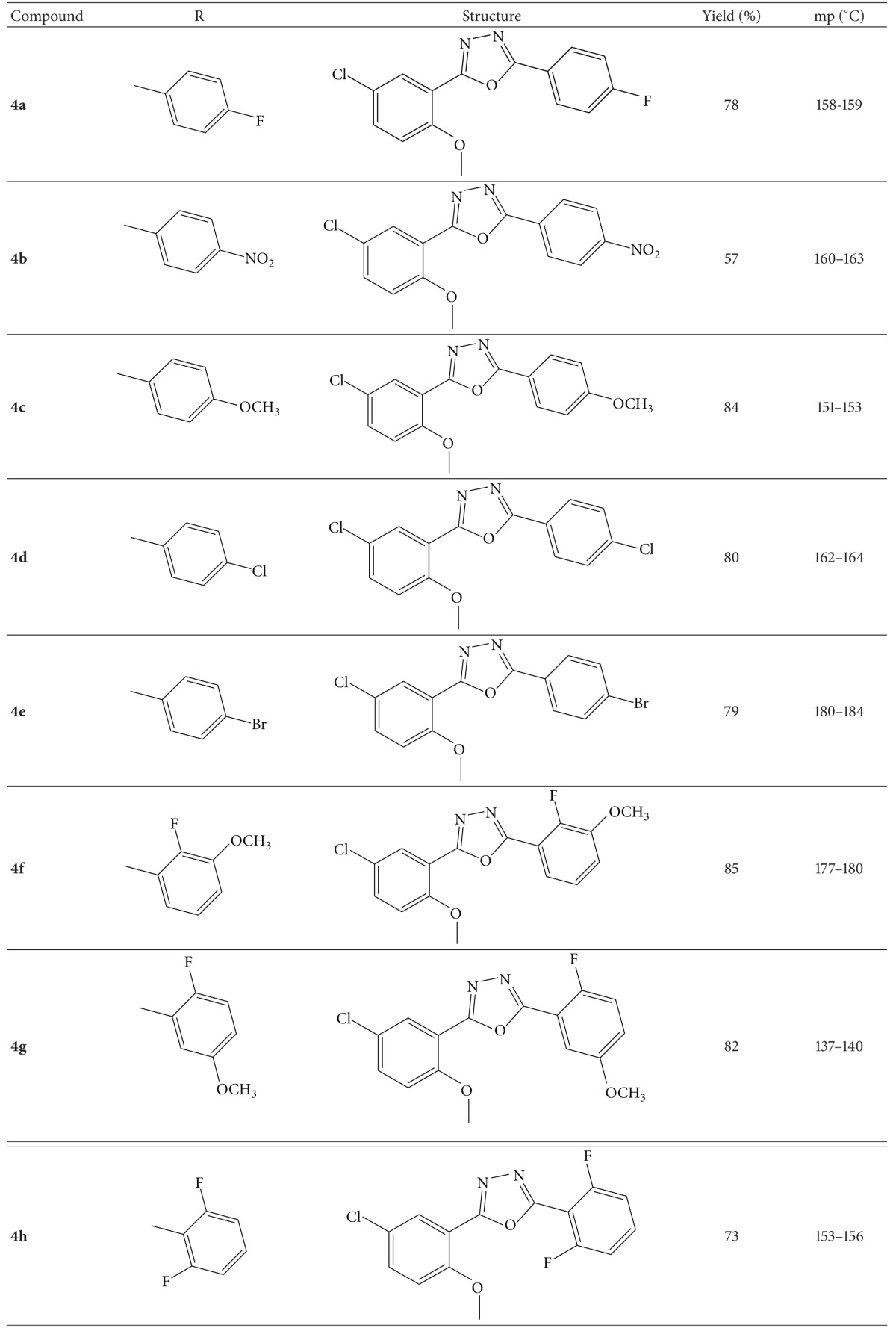

**Table 2:** *In vitro* antimicrobial activity of 1,3,4-oxadiazoles **4**(**a**–**h**).

Compound	Zone of inhibition in diameter (mm)				Percentage of inhibition
	*B. subtilis *	*S. aureus *	*X. campestris *	*E. coli *	*F. oxysporum *
**4a**	15	17	18	15	64.5
**4b**	16	17	19	17	68.4
**4c**	31	26	30	30	78.1
**4d**	15	16	17	17	63.0
**4e**	14	15	16	16	60.2
**4f**	24	22	23	22	77.3
**4g**	21	20	21	20	75.7
**4h**	20	19	20	18	71.4
Bacteriomycin	—	—	34	—	—
Gentamycin	35	30	—	35	—
Nystatin	—	—	—	—	100

## References

[1] Burger A (2003). *Burger’s Medicinal Chemistry and Drug Discovery*.

[2] Farshori NN, Banday MR, Ahmad A, Khan AU, Rauf A (2010). Synthesis, characterization, and in vitro antimicrobial activities of 5-alkenyl/hydroxyalkenyl-2-phenylamine-1,3,4-oxadiazoles and thiadiazoles. *Bioorganic and Medicinal Chemistry Letters*.

[3] Karegoudar P, Karthikeyan MS, Prasad DJ, Mahalinga M, Holla BS, Kumari NS (2008). Synthesis of some novel 2,4-disubstituted thiazoles as possible antimicrobial agents. *European Journal of Medicinal Chemistry*.

[4] Prakash O, Kumar M, Kumar R, Sharma C, Aneja KR (2010). Hypervalent iodine(III) mediated synthesis of novel unsymmetrical 2,5-disubstituted 1,3,4-oxadiazoles as antibacterial and antifungal agents. *European Journal of Medicinal Chemistry*.

[5] El-Emam AA, Al-Deeb OA, Al-Omar M, Lehmann J (2004). Synthesis, antimicrobial, and anti-HIV-1 activity of certain 5-(1-adamantyl)-2-substituted thio-1,3,4-oxadiazoles and 5-(1-adamantyl)-3-substituted aminomethyl-1,3,4-oxadiazoline-2-thiones. *Bioorganic and Medicinal Chemistry*.

[6] Kucukguzel SG, Oruc EE, Rollas S, Sahin F, Ozbek A (2002). Synthesis, characterization and biological activity of novel 4-thiazolidinones, 1,3,4-oxadiazoles and some related compounds. *European Journal of Medicinal Chemistry*.

[7] Kagthara PR, Shah NS, Doshi RK, Parekh HH (1999). Synthesis of 2,5-disubstituted 1,3,4-oxadiazoles as biologically active heterocycles. *Indian Journal of Chemistry B*.

[8] Mohd A, Javed SA, Kumar H (2007). Synthesis of some 1,3,4-oxadiazole derivatives as potential anti-inflammatory agents. *Indian Journal of Chemistry B*.

[9] Akhter M, Husain A, Azad B, Ajmal M (2009). Aroylpropionic acid based 2,5-disubstituted-1,3,4-oxadiazoles: synthesis and their anti-inflammatory and analgesic activities. *European Journal of Medicinal Chemistry*.

[10] Zarghi A, Tabatabai SA, Faizi M (2005). Synthesis and anticonvulsant activity of new 2-substituted-5-(2-benzyloxyphenyl)-1,3,4-oxadiazoles. *Bioorganic and Medicinal Chemistry Letters*.

[11] Kumar D, Sundaree S, Johnson EO, Shah K (2009). An efficient synthesis and biological study of novel indolyl-1,3,4-oxadiazoles as potent anticancer agents. *Bioorganic and Medicinal Chemistry Letters*.

[12] Abdel-Aziz AAM, El-Azab AS, El-Subbagh HI, Al-Obaid AM, Alanazi AM, Al-Omar MA (2012). Design, synthesis, single-crystal and preliminary antitumor activity of novel arenesulfonylimidazolidin-2-ones. *Bioorganic and Medicinal Chemistry Letters*.

[13] Mullican MD, Wilson MW, Connor DT, Kostlan CR, Schrier DJ, Dyer RD (1993). Design of 5-(3,5-Di-tert-butyl-4-hydroxyphenyl)-1,3,4-thiadiazoles, -1,3,4-oxadiazoles, and -1,2,4-triazoles as orally-active, nonulcerogenic antiinflammatory agents. *Journal of Medicinal Chemistry*.

[14] Khan MSY, Khan RM, Drabu S (2001). Anticonvulsant and antibacterial activity of some new 1,3,4-oxadiazole derivatives. *Indian Journal of Heterocyclic Chemistry*.

[15] Liszkiewicz H, Kowalska MW, Wietrzyk J, Opolski A (2003). Synthesis and anti-proliferative activity in vitro of new 5-(2-amino-3-pyridyl)-2-thioxo-3H-1,3,4-oxadiazole derivatives. *Indian Journal of Chemistry B*.

[16] Zheng X, Li Z, Wang Y (2003). Syntheses and insecticidal activities of novel 2,5-disubstituted 1,3,4-oxadiazoles. *Journal of Fluorine Chemistry*.

[17] Przytycka (1954). Synthesis of 2-methoxy-5-chlorobenzoic acid and its ethyl ester. *Roczniki Chemistry*.

[18] Jiang QQ, Darhkijav B, Liu H, Wang F, Li Z, Jiang YB (2010). Anion binding of *N*-(o-Methoxybenzamido)thioureas: contribution of the intramolecular hydrogen bond in the *N*-benzamide moiety. *Chemistry*.

[19] Chandrakantha B, Shetty P, Nambiyar V, Isloor N, Isloor AM (2010). Synthesis, characterization and biological activity of some new 1,3,4-oxadiazole bearing 2-flouro-4-methoxy phenyl moiety. *European Journal of Medicinal Chemistry*.

[20] Bauer AW, Kirby WM, Sherris JC, Turck M (1966). Antibiotic susceptibility testing by a standardized single disk method. *American Journal of Clinical Pathology*.

[21] Satish S, Mohana DC, Raghavendra MP, Raveesha KA (2007). Antifungal activity of some plant extracts against important seed borne pathogens of *Aspergillus* sp. *Journal of Agriculture Technology*.

